# Fibronectin as a Marker of Disease Severity in Critically Ill COVID-19 Patients

**DOI:** 10.3390/cells11091566

**Published:** 2022-05-06

**Authors:** Anna Lemańska-Perek, Dorota Krzyżanowska-Gołąb, Barbara Dragan, Maciej Tyszko, Barbara Adamik

**Affiliations:** 1 Department of Chemistry and Immunochemistry, Wroclaw Medical University, M. Sklodowskiej-Curie 48/50, 50-369 Wroclaw, Poland; dorota.krzyzanowska-golab@umw.edu.pl; 2 Clinical Department of Anesthesiology and Intensive Therapy, Wroclaw Medical University, Borowska 213, 50-556 Wroclaw, Poland; barbara.dragan@umw.edu.pl (B.D.); maciej.tyszko@student.umw.edu.pl (M.T.); barbara.adamik@umw.edu.pl (B.A.)

**Keywords:** COVID-19, fibronectin, intensive care, mortality prediction, biomarkers

## Abstract

The SARS-CoV-2 virus alters the expression of genes for extracellular matrix proteins, including fibronectin. The aim of the study was to establish the relationship between different forms of fibronectin, such as plasma (pFN), cellular (EDA-FN), and proteolytic FN-fragments, and disease severity and mortality of critically ill patients treated in the intensive care unit. The levels of pFN, EDA-FN, and FN-fragments were measured in patients with a viral (N = 43, COVID-19) or bacterial (N = 41, sepsis) infection, using immunoblotting and ELISA. The level of EDA-FN, but not pFN, was related to the treatment outcome and was significantly higher in COVID-19 Non-survivors than in Survivors. Furthermore, EDA-FN levels correlated with APACHE II and SOFA scores. FN-fragments were detected in 95% of COVID-19 samples and the amount was significantly higher in Non-survivors than in Survivors. Interestingly, FN-fragments were present in only 56% of samples from patients with bacterial sepsis, with no significant differences between Non-survivors and Survivors. The new knowledge gained from our research will help to understand the differences in immune response depending on the etiology of the infection. Fibronectin is a potential biomarker that can be used in clinical settings to monitor the condition of COVID-19 patients and predict treatment outcomes.

## 1. Introduction

Coronavirus disease 2019 (COVID-19) is an infectious disease caused by severe acute respiratory syndrome coronavirus 2 (SARS-CoV-2) [1]. The first known case was identified in Wuhan, China, in December 2019, and in the following weeks the disease appeared in other parts of the world. Most COVID-19 patients suffer primarily from respiratory infections; however, clinical deterioration to a more severe and highly fatal form of the disease, including acute respiratory distress syndrome, acute kidney injury, sepsis, shock and multi-organ dysfunction, has been observed in some patients [2,3].

Before the outbreak of the pandemic, the number of patients with a viral infection in intensive care units did not exceed 2.9%, but COVID-19 substantially contributed to an increase in this number and caused a high demand for intensive care services, mainly for advanced respiratory support. [4]. A recent systematic review showed that ICU mortality from SARS-CoV-2 is close to 42% and is higher than usually seen in ICU admissions with other viral pneumonias [5]. Complications in critically ill COVID-19 patients are common and usually associated with pathophysiological changes, such as alveolar macrophage activation [6], lymphopenia [7], excessive production of cytokines [8], micro-thrombosis, and intravascular coagulation [9]. There is an urgent need for sensitive and specific biomarkers related to the viral pathogenesis mechanisms, as well as cell and organ damage, that could be used to monitor the severity of COVID-19.

Our previous study showed that fibronectin (FN), which is a key component of the extracellular matrix (ECM) and glycoprotein that appears at high concentrations in blood, is associated with different kinds of inflammatory diseases [10,11,12]. Different forms of fibronectin have been described: plasma (pFN), cellular (EDA-FN), and proteolytic degradation products (FN-fragments). Fibronectin is involved in various processes, including vascular development, wound healing, and ECM remodeling. Additionally, FN, as a target for many bacterial proteins, contributes to the bacterial colonization of endothelial and epithelial cells [13,14]. Our last study showed that pFN was significantly lower in severe bacterial infections than in control samples from healthy adults, while EDA-FN was significantly higher. Moreover, changes in FN reflected the severity of the bacterial infection: the pFN level in Survivors was significantly higher and the EDA-FN was lower than in Non-survivors [12]. However, little is known about the role and kinetics of FN in the course of viral infections. The data of Xu and co-workers (2020) showed that FN, as an extracellular matrix protein, is involved in interactions with the angiotensin-converting enzyme (ACE)2, the cellular receptor of SARS-CoV-2 [15]. A recent study by Overmyer et al. identified 219 molecules that were strongly correlated with COVID-19 status and severity, including fibronectin [16]. It was shown that the level of EDA-FN, but not pFN, was higher in COVID-19 patients. This finding is important, considering that over-expression of EDA-FN in an animal model of acute ischemic stroke is associated with increased thrombosis and inflammation [17].

There are no studies assessing the relationship between the various forms of FN and the clinical status of patients infected with SARS-CoV-2.

Our study was undertaken to establish the relationship between different forms of FN (pFN, EDA-FN, and FN-fragments) and the severity of the disease and mortality of COVID-19 patients. Considering the high mortality and lack of effective therapies we focused on critically ill COVID-19 patients treated at the intensive care unit (ICU). We also aimed to investigate the differences in the various forms of FN between patients with COVID-19 infections and critically ill patients with bacterial infections. We hypothesized that FN could serve as a potential biomarker useful in a clinical setting for predicting treatment outcomes in the ICU. We expected that there would be differences in the FN levels depending on the etiology of the infection.

## 2. Materials and Methods

### 2.1. Study Group

This was a prospective and observational study; blood samples were collected from patients diagnosed with a COVID-19 infection admitted to the Intensive Care Unit (ICU) between November 2020 and March 2021. A SARS-CoV2 infection was confirmed in each patient on admission to the ICU by a real-time reverse-transcriptase polymerase chain reaction (RT-PCR) assay of nasal or pharyngeal swab probes. Additionally, blood samples from patients with a severe bacterial infection and diagnosis of sepsis on admission to the ICU were collected as a control group. The study was accepted by the Bioethics Committee of Wroclaw Medical University (consent No. 736/2020 and 205/2022). Informed consent was obtained from patients or a patient representative. The study protocol complies with the 1975 Declaration of Helsinki, as revised in 1983. The clinical condition of the patients was assessed with the Acute Physiology and Chronic Health Evaluation (APACHE) II score on admission to the ICU and with the Sequential Organ Failure Assessment (SOFA) score on admission to the ICU and on the third and fifth days of the study. In the ICU, the APACHE II score is widely used as a prognostic tool and the SOFA score is used to monitor the severity of clinical condition and treatment effectiveness (changes in respiratory and cardiovascular parameters, liver and kidney function, activation of coagulation, and patient consciousness).

All analyses of the various forms of FN were performed at the Department of Chemistry and Immunochemistry of Wroclaw Medical and the detailed laboratory protocol is described below.

### 2.2. Blood Sample Collection and Fibronectin Concentration Measurements

Blood samples (2.7 mL, 3.2% sodium citrate as anticoagulant) were obtained from patients suffering from sepsis, septic shock, and COVID-19 upon admission to the ICU and on days 3 and 5. Plasma was immediately separated by centrifugation (2000 rpm, 10 min), and kept at −70 °C until measuring fibronectin.

#### 2.2.1. PFN Level

The level of pFN was measured by an enzyme-linked immunosorbent assay (ELISA), using a well-defined domain-specific monoclonal antibody directed to the central cell-binding domain of FN (FN30-8; M010 TaKaRa Shuzo Co. Ltd., Shiga, Japan), as previously described [18]. The monoclonal anti-FN antibodies were used as a coating agent. The amount of FN bound by the monoclonal antibody was quantified using rabbit anti-FNpolyclonal antibodies (Sigma Chemical Co., St. Louis, MO, USA) and peroxidase-conjugated goat anti-rabbit immunoglobulins (Sigma Chemical Co., St. Louis, MO, USA) as the secondary antibodies. Each sample was analyzed twice and the concentration of pFN was calculated from the calibration curve; the mean value of the two readings was used for the statistical analysis. The pFN concentration was detected with a Synergy LX Multi-Mode Microplate Reader (BioTek, Winooski, VT, USA). A human pFN preparation (Sigma, St. Louis, MO, USA) was used as a standard for determining the FN-ELISA.

#### 2.2.2. EDA-FN Level

The level of EDA-FN was determined in plasma samples using a human cellular fibronectin ELISA kit (Human cFN ELISA Kit, Wuhan Fine Biotech Co., Ltd., Wuhan, China). Each sample was analyzed twice and the concentration was calculated from the calibration curve prepared according to the manufacturer’s protocol. The mean value of the two readings was used for statistical analysis. A Synergy LX Multi-Mode Microplate Reader was used to measure EDA-FN (BioTek, Winooski, VT, USA).

#### 2.2.3. FN-Fragments

On the basis of Western blots, the presence and the quantity of FN-fragments was assessed. Plasma samples containing 300 ng of FN were subjected to SDS-(7.5%) polyacrylamide gel electrophoresis under reducing conditions. Separated proteins were blotted onto nitrocellulose (SERVA Electrophoresis GmbH, Heidelberg, Germany) and incubated with rabbit anti-FN antibody (Sigma Chemical Co., St. Louis, MO, USA) at the dilution of 1:5000. Anti-rabbit IgG HRP-conjugated antibody (Sigma Chemical Co., St. Louis, MO, USA) was applied as a secondary antibody at the dilution of 1:5000. Blots were photographed and analyzed with Gene Tools from the Syngene program.

### 2.3. Statistical Analysis

All statistics were calculated with Statistica 13 software (StatSoft, Inc., Tulsa, OK, USA). The distribution was not normal based on the Shapiro-Wilk test and nonparametric tests were used. Continuous variables were expressed as the median and interquartile range between the 25th and 75th percentiles, whereas categorical variables were expressed as frequencies with percentages. The Mann–Whitney U test was used for comparison of the continuous variables between study groups. The Friedman repeated-measures ANOVA on ranks with the post-hoc test was used to analyze changes in the course of fibronectin over time. Correlations between parameters were tested using Spearman’s test. A *p*-value less than 0.05 was considered statistically significant.

## 3. Results

Samples from 43 consecutive COVID-19 patients with RT-PCR confirmed SARS-CoV2 infection were analyzed. Patients were classified according to their survival status into two study groups: Non-survivors (N = 29, 67%) and Survivors (N = 14, 33%). The severity of the patient’s status on admission to the ICU was determined on the basis of clinical scores, and Non-survivors were characterized by having a more severe clinical condition, as indicated by a worse APACHE II (18 vs. 13 points, *p* = 0.027) and SOFA score (10 vs. 8.8 points, *p* = 0.044) than Survivors. The baseline characteristics of COVID-19 patients are presented in Table 1.

In order to compare pFN and EDA-FN between cases with a viral (COVID-19) or bacterial infection, additional blood samples from 41 ICU patients with a severe bacterial infection were collected. All patients in the latter group had a microbiologically confirmed infection (58% with a Gram-negative and 42% a Gram-positive pathogen) and were diagnosed with sepsis according to the Sepsis-3 definition on admission to the ICU [19]. The median APACHE II score in this group was 25 points (IQR 20.0–28.0) and the median SOFA score was 10 points (IQR 8.0–11.0) on admission to the ICU. The indices of an inflammatory response recorded on admission to the ICU were high: procalcitonin 9.0 ng/L (IQR 2.7–20.2), c-reactive protein 194.3 mg/L (IQR 98.7–305.2), and white blood cell count of 15.8 × 103/µL (IQR 10.4–21.7). Out of 41 patients, 18 died (44%) and 23 survived (56%).

### 3.1. The Concentration of pFN and EDA-FN in Patients with COVID-19

**Figure 1:**
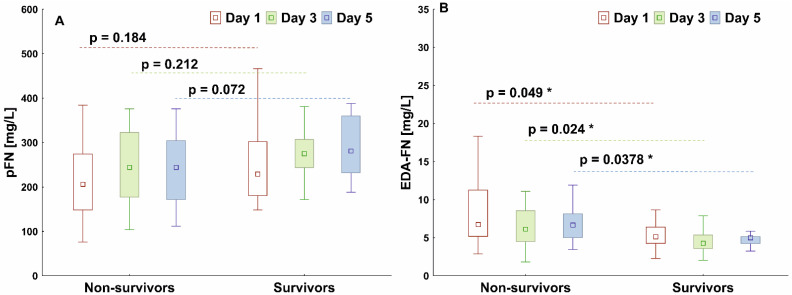
Graph comparing plasma fibronectin (pFN, (**A**)) and cellular fibronectin (EDA-FN, (**B**)) concentrations of COVID-19 Non-survivors and Survivors. The box plots represent the median values (midpoint) with interquartile range between the 25th and 75th percentiles (box); the whiskers represent the minimum and maximum values. * indicates *p*-value less than 0.05.

The median pFN concentration measured on day 1 was higher in Survivors than in the Non-survivors and increased continuously over the following days of observation, but the differences between groups were not statistically significant (Figure 1A). The median value of the EDA-FN concentration was significantly higher in the Non-survivors than in the Survivors (*p* = 0.049) at each observation day (Figure 1B).

Differences in pFN and EDA-FN between the specified time points (days 1, 3 and 5) were analyzed in each group using a Friedman ANOVA with post-hoc tests. There were no differences between the specified time points for the pFN (Non-survivors: *p* = 0.157, Survivors: *p* = 0.442). There were no differences between the specified time points for the EDA-FN in the Non-survivors (*p* = 0.662), but in the Survivors the EDA-FN concentration decreased significantly (*p* < 0.05) on days 3 and 5 compared to the baseline.

### 3.2. Comparison of pFN and EDA-FN Concentrations in Patients with Viral and Bacterial Infections

**Figure 2:**
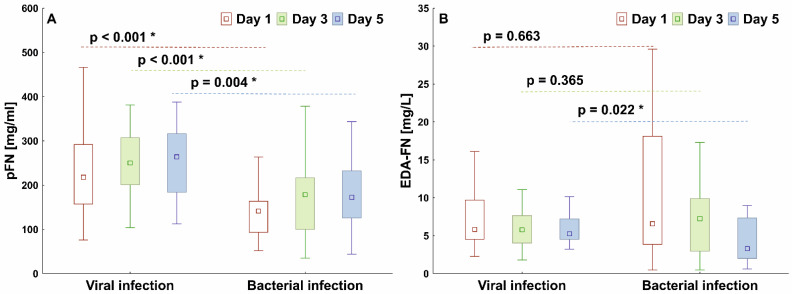
Graphs comparing plasma pFN (**A**) and EDA-FN (**B**) concentrations of patients with a viral infection (COVID-19) or bacterial infection (sepsis). The box plots represent the median values (midpoint) with interquartile range between the 25th and 75th percentiles (box); the whiskers represent the minimum and maximum values. * indicates *p*-value less than 0.05.

The median pFN concentration in viral infections (COVID-19 patients) was significantly higher than in bacterial infections (septic patients) on each study day, while the median EDA-FN concentration was higher in viral than in bacterial infections only at the end of observation (Figure 2).

### 3.3. Correlations between Fibronectin and Clinical Scores

On ICU admission, a significant correlation between the APACHE II score and EDA-FN was recorded (r = 0.41, *p* = 0.006). Also, the SOFA scores calculated on days 1, 3, and 5 correlated with the corresponding EDA-FN values (Table 2). There were no correlations between the pFN and the severity scores on any of the study days.

### 3.4. FN-Fragments

**Figure 3:**
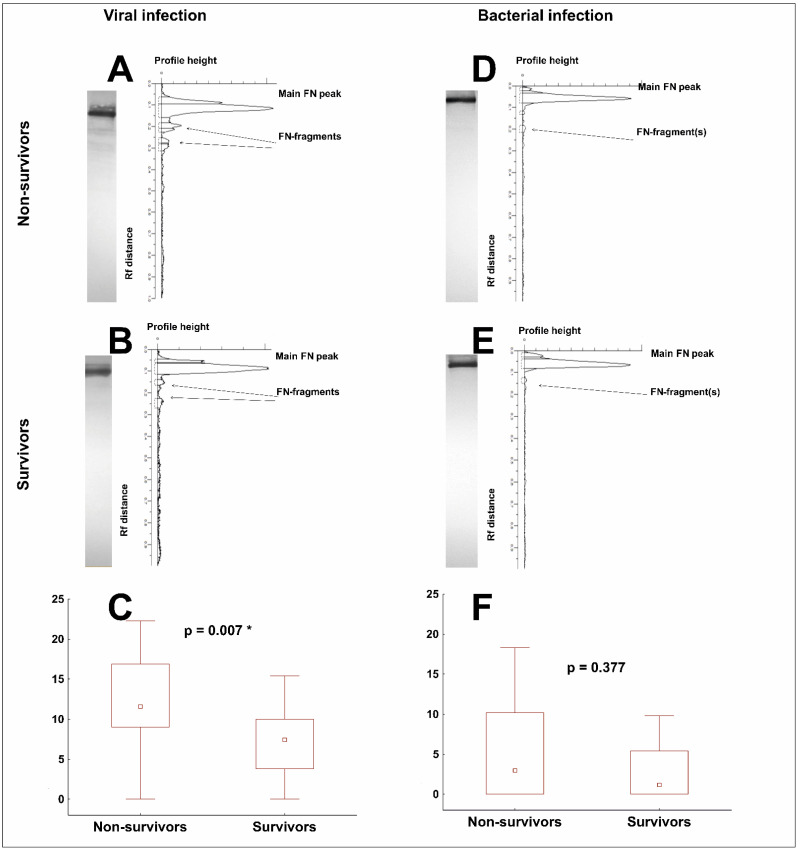
The occurrence of FN-fragments in the plasma of patients with a viral infection (COVID-19, left panel) or a bacterial infection (sepsis, right panel). Representative immuno-patterns of FN and FN-fragments and the corresponding densitograms for Non-survivors (graphs (**A**,**D**)) and for Survivors (graphs (**B**,**E**). Correspondingly, graphs (**C**,**F**) compare the number of FN fragments, expressed as a percentage of all FN forms detected in the electrophoretic pathway between Non-survivors and Survivors. The box plots represent the median values (midpoint) with interquartile range between the 25th and 75th percentiles (box); the whiskers represent the minimum and maximum values. * indicates *p*-value less than 0.05.

The occurrence of FN-fragments was confirmed by Western blotting with anti-FN polyclonal antibodies after SDS-(7.5%) polyacrylamide gel electrophoresis under reducing conditions. An estimation of the quantity of FN-fragments in the lane on the immunoblot was performed by calculating the percentage of the area under the peaks corresponding to the bands identified as FN-fragments in the total area under the densitomeric curve (Figure 3). For statistical analysis, the sum of all peaks corresponding to the FN-fragments was taken.

Firstly, we identified the FN-fragments in the plasma of patients with COVID-19 in 95% of the samples (41 out of 43 tested). The quantity of FN-fragments was significantly higher in the plasma of Non-survivors than of the Survivors (11.6 vs. 7.5, *p* = 0.007) (Figure 3C).

Then, we compared the occurrence of FN-fragments between the COVID-19 and bacterial sepsis samples. Interestingly, proteolytic degradation of FN was more frequent in the plasma of the COVID-19 patients than in the plasma of patients with bacterial sepsis. The FN-fragments were present in only 56% of samples from patients with bacterial sepsis (19 out of 34 tested) and there were no statistically significant differences between patients who died and those who survived (3.0 vs. 1.2, *p* = 0.377) (Figure 3F).

## 4. Discussion

In this study, we analyzed the changes in two forms of fibronectin, pFN and EDA-FN, associated with the severity of the clinical condition of COVID-19 patients treated in the ICU. Our results indicate that the level of EDA-FN, but not pFN, was related to the patient’s treatment outcome and was significantly higher in patients who died, than in survivors. Also, the proteolytic degradation of FN was ubiquitous and related to the survival of COVID-19 patients. The second aim of the study was to investigate differences in the levels of various forms of FN in viral and bacterial infections. A well-defined group of ICU patients diagnosed with bacterial sepsis was compared to patients with COVID-19. We found that the pFN form was significantly higher throughout the study and the EDA-FN form was higher at the end of observation in viral infections compared to bacterial infections. Also, the proteolytic degradation fragments of FN were present in a majority of COVID-19 patients and only in about half of the cases of bacterial sepsis. FN, as an extracellular matrix protein, is involved in the interactions with the angiotensin-converting enzyme (ACE)2, the cellular receptor of the SARS-CoV-2; hence the high level of FN and the ubiquitous presence of FN- fragments indicate an extended, and significant, reorganization of the extracellular matrix associated with COVID-19. To our best knowledge, this is the first study to show an association between the levels of various forms of FN and the outcome of ICU patients with COVID-19.

Previously, EDA-FN was shown to be undetectable or very low in healthy adults, but increased significantly in various pathological conditions, including sepsis [12,20]. A recent large-scale, multi-omnic analysis [16] showed that the SARS-CoV-2 virus induced upregulation of the EDA-FN form in the general population (both hospitalized and not hospitalized) of COVID-19 patients compared to patients without COVID-19. In our study, an abundance of FN forms was found in the most critically ill patients with COVID-19 who required ICU treatment. We showed that the EDA-FN form was present in all COVID-19 patients with levels significantly higher in those who eventually died. Moreover, the EDA-FN strongly correlated with the severity of the clinical condition of the patients, assessed with the routinely used ICU clinical scales APACHE II and SOFA: the worse a patient’s clinical condition, the higher the APACHE II and SOFA scores and the higher the level of the EDA-FN form. This form of FN promotes inflammatory processes by activating the Toll-like-receptor-4 signaling pathway and is a ligand for integrin-91, which is expressed by numerous inflammatory cells, including neutrophils and macrophages [17]. Data from Dhanesha and co-workers showed that the over-expression of EDA-FN in mice was associated with increased thrombosis and inflammation. Therefore, elevated levels of EDA-FN in COVID-19 may be associated with the emergence of micro-thrombosis, which contributes to vascular occlusion and organ ischemia. As a consequence, severe organ damage can be observed in ICU patients with COVID-19, as evidenced by the high SOFA scores also recorded in our study. Additionally, high levels of EDA-FN may derive from a damaged or repaired extracellular matrix, and indeed, we have observed the presence of FN proteolytic degradation products in nearly all COVID-19 patient samples. The quantity of FN-fragments was significantly greater in patients who eventually died. This observation was characteristic of the COVID-19 infections, because although we also observed the proteolytic degradation of FN in bacterial sepsis, it was less frequent, less intense and unrelated to the severity of the patient’s condition [12].

The pFN was also measured in blood samples from COVID-19 patients and compared with the results recorded in the group of patients diagnosed with bacterial sepsis; the pFN form was much higher in viral than in bacterial infections. This finding confirms our previously published data showing a significant reduction in pFN levels in severe bacterial infections. We hypothesized that the activation of coagulation in sepsis might be responsible for the decrease in pFN levels, since the plasma form of FN is a crucial component of the fibrin clot [12]. Several previously published studies have shown that the pFN level in healthy adults is approximately 300 mg/L [11,18,20,21]. In this study, pFN levels measured in COVID-19 patients were similar to those previously reported in healthy adults. In addition, changes in pFN recorded during the study were not related to the patient’s condition, as no marked differences were observed between the Survivors and the Non-survivors. However, a significantly higher level of pFN in COVID-19 patients, than in patients with sepsis, may indicate no pFN loss during a viral infection or might be the result of overproduction of FN caused by the viral infection. Such overproduction was observed by Ren et coworkers in patients with hepatitis B infection, with the levels of FN more than twice as high as in healthy subjects [22]. In addition, this study demonstrated that HBV expression increased both FN mRNA and protein levels in the in vitro hepatic cell line cultures. Downregulation of FN mRNA inhibited HBV DNA replication and protein synthesis by stimulating the production of endogenous type I interferon-α [22]. Thus, the observed levels of pFN in COVID-19 patients may be the result of two processes: overproduction and loss of FN existing at the same time. Both of these hypotheses require additional research.

Numerous studies have been carried out to develop diagnostic tools to distinguish viral from bacterial infections [23,24,25]. The results of these studies could be useful in identifying biomarkers that could be useful in a clinical setting to distinguish between bacterial and viral infections and to predict treatment outcome. The differences in the level of individual forms of fibronectin observed in our study between the COVID-19 group and the bacterial sepsis group indicate that there are significant differences in the activation of immune response and the importance of fibronectin in viral and bacterial infections. A recent study of SARS-CoV-2 entry into target cells found that the virus alters gene expression, including those encoding fibronectin, a tissue inhibitor of metalloproteinase, angiotensinogen, a transforming growth factor beta, and the vascular endothelial growth factor in alveolar epithelial cells [15]. Similar changes were also seen in the lung tissues in pulmonary fibrosis, suggesting that SARS-CoV-2 infection contributes to the progress of pulmonary fibrosis. Fibronectin is a key extracellular matrix protein, involved in the processes of remodeling damaged tissues [20] and fibrosis [26]. In critically ill patients infected with SARS-CoV-2, the expression of extracellular matrix proteins, including fibronectin, is significantly elevated in alveolar epithelial cells, indicating a greater risk of developing lung fibrosis [15].

It should be noted that from the beginning of the COVID-19 pandemic, many potential biomarkers of disease severity in critically ill patients have been proposed. Multiple studies have shown that the clinical course of critically ill COVID-19 patients is associated with elevated levels of routine laboratory parameters such as WBC, creatinine, C-reactive protein, procalcitonin, as well as with lymphopenia and thrombocytopenia [27]. It has also been shown that many biomarkers which are not routinely monitored in the hospital laboratory can be useful for COVID-19 patients in identifying high-risk cohorts, assessing responses to new therapies, and predicting treatment outcome. KL-6 (Krebs von den Lungen-6), one of the mucins expressed in the lung cells, has been proposed as a marker of the severity of lung damage caused by SARS-CoV-2 [28]. Previously, the usefulness of KL-6 was confirmed in fibrotic interstitial lung disease and acute respiratory distress syndrome [29]. Multiple studies have shown that hiperferritinemia is often associated with viral infections and related to poor treatment outcome of COVID-19 patients [30,31]. In our study, baseline ferritin was above the reference range, but there was no significant difference between Survivors and Non-survivors. The immune response in COVID-19 is characterized by increased secretion of pro-inflammatory cytokines, and interleukin 6, one of the major pro-inflammatory cytokines, has been proposed to be a marker of severity in COVID-19 patients. The recently published meta-analysis by Mojtabavi et al. showed that the level of IL-6 measured in the blood can be a reliable indicator of the severity of the disease in patients with severe or critical symptoms of COVID-19 [32]. In our study, a large variation in IL-6 results was observed at baseline, and there was no significant difference between Survivors and Non-survivors. Despite advances in understanding the importance of biomarkers in predicting COVID-19 outcomes, future research is needed to find an algorithm that can be used in a clinical setting to monitor the status of COVID-19 patients.

This study has several limitations. Although we analyzed different forms of fibronectin involved in the activating of the extracellular matrix response, we did not measure the FN-fibrin complexes that may arise during activation of the inflammatory response. In addition, we analyzed the proteolytic degradation products of FN present in the blood, but we were unable to determine the degree of proteolytic degradation for each form, i.e., pFN or EDA-FN. Our study was conducted in a single center, which presents a challenge to generalizing our findings. These are preliminary data with interesting results that should encourage further patient recruitment in a multi-center study.

## 5. Conclusions

Our findings are of clinical importance as the SARS-CoV-2 virus alters the expression of genes for extracellular matrix proteins, including fibronectin. The new knowledge gained from our research will help to understand the differences in immune responses in sepsis, depending on the etiology of the infection, which could help in the development of future immunomodulatory drugs. Recent analyses of data from multilevel proteomics show that there are significant differences in immune responses that can be used to distinguish viral from bacterial sepsis [16,33]. We found that the kinetics of changes in individual forms of FN differed significantly between viral and bacterial infections. Fibronectin is a potential biomarker that can be used in a clinical setting to monitor the condition of COVID-19 patients and predict treatment outcomes in the ICU. Nevertheless, more research is needed into the role of extracellular matrix proteins in the pathogenesis of COVID-19.

## Figures and Tables

**Table 1:** Characteristics of patients with a COVID-19 diagnosis on admission to the ICU.

	Non-Survivors	Survivors	*p* Value
	*n* = 29	*n* = 14	
Age	62.0 (53.0–69.0)	53.5 (40.0–60.0)	0.029 *
Female/Male	7/22	6/8	
APACHE-II score	18.0 (12.0–27.0)	13.0 (9.0–15.0)	0.027 *
SOFA score	10.0 (8.0–12.0)	8.5 (7.0–9.0)	0.044 *
Medical history *n* (%)			
Hypertension	17 (59)	6 (43)	0.331
Heart failure	6 (21)	0	0.022 *
Diabetes	6 (21)	1 (7)	0.254
Asthma	2 (7)	1 (7)	0.703
COPD	1 (3)	0	0.674
Cancer	4 (14)	0	0.192
Stroke	4 (14)	0	0.192
Kidney failure	3 (11)	0	0.296
Pregnancy	0	2 (14)	0.100
Obesity	4 (14)	3 (21)	0.409
Smoking	6 (21)	0	0.779
Procalciton [ng/L]	0.57 (0.15–2.1)	0.25 (0.1–0.6)	0.167
C-reactive protein [mg/L]	122.0 (53.0–216.0)	129.5 (79.0–159.0)	0.888
White blood cells [10^3^/µL]	14.9 (11.7–17.8)	13.7 (12.0–22.4)	0.888
Ferritin [ng/mL]	1112 (446–1994)	1492 (704–1725)	0.620
Intereukin 6 [pg/mL]	77.6 (20.5–700.0)	37.1 (9.6–163.5)	0.173
LOS ICU [days]	16 (9–22)	14 (10–25)	0.747
LOS hospital [days]	19 (11–26)	22 (13–28)	0.509

APACHE II, Acute Physiology and Chronic Health Evaluation II; SOFA, Sequential Organ Failure Assessment; ICU, intensive care unit; LOS, length of stay. The *p*-value represents differences between the groups; * indicates *p*-value less than 0.05.

**Table 2:** Spearman’s rank correlation coefficient between pFN, EDA-FN and the APACHE II score calculated on admission to the ICU and between pFN, EDA-FN and the SOFA score calculated on admission, day 3 and 5.

Day	APACHEII		APACHEII		SOFA		SOFA	
	& pFN		& EDA-FN		& pFN		& EDA-FN	
	r	*p*	r	*p*	r	*p*	r	*p*
1	−0.26	0.086	0.41	0.006 *	−0.14	0.365	0.42	0.005 *
3					−0.13	0.418	0.62	<0.000 *
5					−0.19	0.303	0.56	0.001 *

r—correlation coefficient; *p*—statistical significance of correlation; * indicates *p*-value less than 0.05.

## Data Availability

The data presented in the study are available on the request from the corresponding author. The data have not been made publicly available because they contain information that could compromise the privacy of the study participants.
